# Cryo‐EM structures of Fab‐bound CHIP E3 ligase reveal conformational switching and dual roles in regulation of tau accumulation

**DOI:** 10.1002/alz70855_103348

**Published:** 2025-12-23

**Authors:** Aparna Unnikrishnan, Dong hee Chung, Emily J Connelly, Aye Thwin, Shweta Devi, Jason E. Gestwicki, Charles S Craik, Daniel Southworth

**Affiliations:** ^1^ University of California San Francisco, San Francisco, CA, USA

## Abstract

**Background:**

E3 ligase CHIP interacts with tau directly and via chaperones in Alzheimer's disease and other tauopathies. These interactions regulate tau turnover by facilitating its ubiquitination and degradation, and by suppressing tau aggregation. Modulating CHIP‐driven clearance mechanisms offers a promising therapeutic strategy. However, the structural basis for CHIP's activity remains unclear. Developing and characterizing antigen‐binding antibody fragments (Fabs) that can bind unique 3D epitopes on CHIP can facilitate Cryo‐EM structural studies, provide insights into CHIP‐mediated tau clearance, and explore their therapeutic potential.

**Method:**

Recombinant Fabs were developed to conformation‐selectively target CHIP by bio‐panning and BLI binding assays. Selected Fab interactions that formed stable complexes with CHIP were employed for Cryo‐EM high‐resolution structural studies of CHIP in solution. The Fabs were then functionally screened using *in vitro* ubiquitination activity assays, E2‐binding assays, ThT‐based tau fibrillation assays etc.

**Result:**

Cryo‐EM characterization of multiple Fab‐bound CHIP complexes with different 3D binding epitopes revealed three distinct conformational states of CHIP in solution: asymmetric, intermediate, and symmetric dimers. These states showed changing accessibility for CHIP U‐box‐E2 enzyme interactions by a conformational switch between half‐site‐active asymmetric CHIP and potentially fully active symmetric CHIP dimers. Further, using the Fabs as mechanistic probes, functional analysis revealed potent Fab‐binding dependent‐effects on CHIP's E3 ligase and direct anti‐tau aggregation activities. Fab '2F1' forms a 2:2 complex with CHIP and potently inhibits its ubiquitination activity by directly obstructing the essential E2‐CHIP interactions. Weaker binder Fab 'H1' suppresses ubiquitination and forms both 1:2 and 2:2 complexes with CHIP, significantly enhancing its ability to suppress tau aggregation. Fab '2D2' forms a 1:2 complex with CHIP, blocks CHIP‐tau interactions and stabilizes the CHIP dimerization interface.

**Conclusion:**

Structural and functional screening of Fabs that were generated to specifically target 3D epitopes on CHIP revealed potent modes of CHIP functional modulation. Insights from the corresponding CHIP‐Fab structures help in better understanding the CHIP‐driven tau clearance mechanisms and in describing unique therapeutic target sites on CHIP.

